# Production of Synthetic Phosphoanhydrite and Its Use as a Binder in Self-Leveling Underlayments (SLU)

**DOI:** 10.3390/ma10080958

**Published:** 2017-08-17

**Authors:** Cecília Ogliari Schaefer, Malik Cheriaf, Janaíde Cavalcante Rocha

**Affiliations:** 1 Post-Graduate Program in Civil Engineering, Federal University of Santa Catarina, CEP 88040-900 Florianópolis, SC, Brazil; cissa.og@gmail.com; 2 Department of Civil Engineering, Federal University of Santa Catarina, CEP 88040-900 Florianópolis, SC, Brazil; malik.cheriaf@gmail.com

**Keywords:** calcium sulfate, phosphogypsum, anhydrite, self-leveling mortars

## Abstract

An experimental study was conducted to investigate the potential use of phosphogypsum (PG) to produce self-leveling underlayments. The study was designed in two stages. Initially a phosphoanhydrite (PA) was produced by heating phosphogypsum at temperatures of 350 °C, 450 °C, 550 °C, and 650 °C. Two periods of heating were applied (2 and 4 h). The formation of anhydrite was determined by thermogravimetric analysis (DTA-TG) and confirmed by X-ray diffraction (XRD). The results show that anhydrite II was obtained at temperatures above 450 °C, and at higher calcination temperatures the PA solubility was lower. In the second stage of this research, the PA was used in self-leveling underlayments as the main binder in the ternary system comprised of calcium sulfate, calcium aluminate cement, and Portland cement. Self-leveling mortar screeds produced using PA (550 °C/4 h) and PA (650 °C/4 h) showed the best performance in terms of mechanical strength and no degradation was observed after immersion and immersion-drying tests. The formation of ettringite, identified by scanning electron microscopy (SEM), may have contributed to these results. Morphological changes were studied using the scanning electron microscopy (SEM) technique.

## 1. Introduction

A self-leveling underlayment (SLU) is a fluid product comprised of a binder, sand, water, and additives, and its main characteristics are low viscosity and high fluidity [1,2]. This building material requires no vibration and leveling, and has a very small thickness of around 30 mm.

Most SLUs are comprised of either Portland and calcium sulfoaluminate cement or calcium sulfate. Cement-based SLUs have better water resistance, higher strength and a shorter curing period than calcium sulfate-based SLUs. The advantages of calcium sulfate-based SLUs are fast setting, low shrinkage, and little cracking and they can be applied to large surfaces without construction joints; however, they may be sensitive to moisture depending on the calcium sulfate used [3,4]. One way to minimize water sensitivity is through the use of calcium aluminate cement (CAC). This type of cement, which is rich in alumina, is normally used in self-leveling underlayments in ternary mixtures of CAC, calcium sulfate (C$), and Portland cement (PC). One use of CAC is as a precursor for the formation of ettringite (C_3_A·3C$·H_32_). These systems are known as ettringite-rich products due to the main hydration product formed. When formulated correctly, ettringite-rich products are able to provide rapid drying and hardening and shrinkage compensation and they are not sensitive to the action of water [5]. 

The authors [6] carried out an extensive investigation on the effect of calcium sulfate hemihydrate on the hydration of ternary blends composed of CAC and a limestone-CAC system was also evaluated. They concluded that high levels of calcium sulfate could promote expansion and lead to the destruction of the material.

However, the products of the hydration of Portland cement (PC-based system), such as C–S–H, can favor the interlocking of the crystals in the matrixes, producing predominantly with calcium sulfate [7,8]. 

In addition, the type of calcium sulfate employed affects the expansion or retraction of the SLU. Beta hemihydrate has expansive as well as dihydrate characteristics, although the latter is on a smaller scale. Anhydrate, in general, shows retraction with or without a little expansion [9]. The formation of a form of ettringite known as massive ettringite is related to expansion and cracking in systems containing beta hemihydrate [10]. Anhydrite forms non-expansive ettringite, with the typical format of long fine needles [11]. Expansion is normally attributed to the crystal growth theory. This suggests that ettringite grows around the cement grain, resulting in a crystallization pressure which leads to expansion. This behavior occurs when the crystals interact with each other [11]. Although some authors have reported the formation and expansion of ettringite, this behavior is still not completely understood. According to literature review, ternary have been blended in CAC-dominate formulations, with CAC/C$ ratios of (2.5–2.8) [12,13], 1.5–1.8 [14], or in a PC-dominated formulation with a CAC/C$ ratio of 2.0–2.8 [7,13,15]. There is formation of an ettringite-phase in both systems, but the CAC-dominated formulations react faster and produce more ettringite than PC-dominated formulations [15].

In recent study, the authors [13] proposed two main stages in hydration processes. The first involves the dissolution of the anhydrous and sulfate phase (calcium sulfate α-hemihydrate), where the sulfate source is totally consumed within 1 day and the formation of ettringite continues for 28 days and then decreases. An AH3 phase appears in system, and suggesting a delay in the silicate reaction due to a barrier layer covering the surface of unhydrated particles. The second stage is related to the formation of monosulfate through the consumption of ettringite. In view of practical purposes and applications, an overview of the properties of ternary mixtures of CAC-PC-C$, based on study of a natural anhydrite and alpha-hemihydrate [16] has been given. 

Calcium sulfate (C$) can be found in anhydrite, hemihydrate, and dehydrate forms, and can be obtained from natural source or byproducts as secondary gypsum. Several papers describe existing forms of calcium sulfate [17,18,19,20,21] that are obtained as a function of temperature and are basically dihydrate or gypsum (CaSO_4_·2H_2_O), hemihydrate or plaster of Paris (CaSO_4_·1/2H_2_O), and anhydrite (CaSO_4_). The hemihydrate is obtained within the temperature range of 140–160 °C and has two forms of crystallization: α and β. The β form is obtained by calcination and α-hemihydrate is derived from the autoclaving process. In the temperature range of 160–250 °C, anhydrite III or soluble anhydrite is formed, an unstable phase which becomes hemihydrate in the presence of moisture. Above 300 °C anhydrite III becomes anhydrite II (CaSO_4_) which has a slower hydration rate, but this can be accelerated by means of activators that enhance the rate of dissolution, forming transient hydration products that act as nucleation points and favor the formation of gypsum (CaSO_4_·2H_2_O) [19]. Some examples of activators are a carbonate component (Li_2_CO_3_), a sulfate-base component (Na_2_SO_4_, NaHSO_4_, FeSO_4_, K_2_SO_4_, KHSO_4_), lime, Ca(OH)_2_ and NaOH, Portland cement clinker, and others [20]. Anhydrite I (CaSO_4_) is obtained only at temperatures of around 1000 °C and may be inert [22,23].

Phosphogypsum or chemical gypsum is a byproduct generated during the production of phosphoric acid used in the correction of farmland. Phosphogypsum may provide a source of calcium sulfate for use in the production of self-leveling underlayments. It is possible to obtain anhydrite from the heat treatment of phosphogypsum, as shown in a study by [23]. According to [9,24,25,26,27], the morphology plays an important role in the crystal growth and mechanical properties of a calcium sulfate-based system. The dissolution rate of calcium sulfate is related to the extent and speed of dissolution in aqueous media. It is possible to evaluate the calcium sulfate dissolution rate by estimating the content dissolved (g/L) in an aqueous medium according to the relationships CaSO_4_^2−^/H_2_O [27]. When anhydrite comes into contact with water, its dissolution starts immediately and leads to a saturated solution of Ca^2+^ and SO_4_^2−^ ions [17]. 

Numerous studies involving the production of phosphogypsum have been carried out, covering phosphogypsum generation and some environmental aspects [28,29]. Trace metal contents (arsenic, silver, barium, cadmium, chromium, lead, mercury, and selenium) in some PG sources, also radionuclides naturally present in the phosphate rock. Initially, the research carried out in Brazil and worldwide was focused on the radioactive species present in phosphogypsum [30,31,32,33] and also, radon exhalation from stockpiles and areas near stacks. The specific activities of the radionuclides ^226^Ra, U, ^232^Th, ^228^Ra, and ^210^Po have been determined at different production sites, and phosphogypsum can be classified as a TENORM (Technologically Enhanced Naturally Occurring Radioactive Material). Recent approaches have focused on the assessment of radionuclides activity concentration in building materials [33,34,35]. To evaluate the radiological impact associated with the use of phosphogypsum, a pilot study was performed to investigate gamma exposure and radon concentration [34]. This approach consisted of developing a prototype using building materials based on phosphogypsum as a secondary raw material, simulating the application of new materials. Internal and external exposure was monitored and the activity concentrations of ^40^K, ^226^Ra, and ^232^Th were considered to establish the *I* index. In the first step, the index is determined via the following equation:

(1) $$I=\frac{a_{Ra-226}}{300BqKg^{-1}}+\frac{a_{Th-232}}{200BqKg^{-1}}+\frac{a_{K-40}}{3000BqKg^{-1}}$$

Due to the variation in the density, this index can be adjusted by an expression, considering the density of the materials and the wall thickness [33,35]. With regard to Brazilian phosphogypsum, the authors verified that the external exposure dose should be <1 mSv y^−^^1^, this being the recommended value for the protection of the public against the radiological impact of naturally occurring radioactive materials [34,36]. 

Research efforts to develop a norm are now in progress; however, data on radon emanation from specific types of building materials are scarce in the literature. According to [35], an additional target must be sought in order to encourage the use of low-radiation construction products as building materials. In a Brazilian study on PG conducted on building dwellers (prototype), it was concluded that the use of phosphogypsum as a building material represents no additional internal exposure risk due to the inhalation of radon and external exposure from gamma emitters [37]. Application (prototype) and modelling approaches can be considered to evaluate random exhalation from building materials and these represent appropriate areas for further studies. 

Thus, the aim of this study was to obtain a synthetic anhydrite (anhydrite II), referred to herein as phosphoanhydrite (PA), from the calcination of phosphogypsum and subsequently to apply it as the main binder in SLUs. The advantages of this type of cement compared to ordinary Portland cement (OPC) are related to reducing the clinker factor and minimizing the amount of Portland cement used, reducing CO_2_ emissions, and introducing a new binder based on calcium sulfate.

## 2. Materials and Methods

### 2.1. Materials

The main raw material used in this study was phosphogypsum, which is a byproduct of phosphoric acid production. Phosphogypsum is a type of calcium sulfate dihydrate processed and marketed as agricultural gypsum, and its calcination when applying different temperature ranges yields different types of calcium sulfate. This study was conducted with the following types of calcium sulfate: dried phosphogypsum (PG REF), β-hemihydrate, and phosphoanhydrite (PA). PG REF represents phosphogypsum dried at a temperature of 105 °C, without treatment. The β-hemihydrate, a commercially available product was donated by a company that sells this product for landfarm application, it was calcined at 190 °C. The PA was obtained from the calcination of the phosphogypsum in a muffle furnace in the temperature range of 350 °C to 650 °C. Two calcination periods were tested: 2 and 4 h. The material was obtained in the form of a fine powder without agglomeration and did not require grinding.

The physical properties of the PA, such as specific gravity, specific surface area, and loss on ignition, were determined and the values obtained are listed in Table 1.

Table 2 shows the results for the chemical analysis of the different calcium sulfate samples studied after calcination for a period of 4 h. For the chemical analysis, tablets of the solid material were prepared and these were analyzed on an energy dispersive X-ray (EDX) spectroscopy. The main constituents were CaO and SO_3_, representing around 70% of the composition.

For the production of the calcium sulfate-based SLUs, two types of cement were used: calcium aluminate cement (CAC) and a Brazilian Portland cement with lime (PC) called CP II-F, strength class 32. The results for the chemical analysis of the cements used are shown in Table 3.

### 2.2. Methods

#### 2.2.1. Analytical Methods

Raw material and filtered solution were analyzed using energy dispersive X-ray fluorescence spectrometry (Model 700 HS, Shimadzu, Tokyo, Japan).

Particle size was determined by laser grain size measurements (Microtrac Model S 3500, Largo, FL, USA).

X-ray diffraction analysis was carried in raw material and in mortar at 28 days using power an X-Pert system (Philips, Almelo, The Netherland), with CuKα = 1.54 Å, step range from 4 to 70°, with a step size 0.022 seg, operated at 40 kV and 40 mA.

The SEM analysis was performed with a Philips microscope (model XL30, Philips, Almelo, The Netherland). Samples are covered with Au.

Thermogravimetric analysis (TGA/DSC) was carried out using STD Q600 (TA Instruments, New Castle, DE, USA), TA instruments, 50 mg of pulverized samples (<75 µm), temperature ranged from ambience to 1000 °C with a heating rate of 5 °C/min under ultrapure N_2_ atmosphere to prevent carbonatation.

#### 2.2.2. Formulations of Self-Leveling Underlayments (SLUs)

In order to assess the behavior of phosphoanhydrite (PA) as a main binder in self-leveling underlayments, a ternary system composed of PA, CAC, and Portland cement (PC II-F) was prepared. In the formulation, the PC acted as an activator of the above-mentioned ettringite-rich system. The same formulation was used for each of the different types of calcium sulfate (PG REF, β-hemihydrate, PA), and the dried phosphogypsum (PG REF) was taken as the reference. The compositions of the formulations are detailed Table 4. For all mixes, the water/binder ratio was maintained constant at 0.6. A polycarboxylate superplasticizer was used in order to obtain the necessary fluidity, and different dosages were required according to the calcination temperature used to obtain the PA. A rheology-stabilizing additive was also used, with a content of 0.03% relative to the total weight of the binder, to avoid the segregation of the mixture. This additive was in powder form, was totally soluble in water, and it increased the viscosity of the mixture.

One of the requirements of these self-leveling underlayments (SLUs) is that they maintain their workability for 180 min. Thus, a retardant was needed to maintain the workability for the required period. Two retardant liquids, citric acid and sodium gluconate, were tested at a concentration of 1.5% relative to the total weight of the binders.

In this article, the nomenclature used for the SLU samples was related to the name of the calcium sulfate used in their production, followed by the calcination temperature. For example, PG REF relates to the SLU produced from phosphogypsum and PA (450 °C/4 h) relates to that produced with phosphoanhydrite obtained at a temperature of 450 °C, applying 4 h of calcination.

#### 2.2.3. Solubility of Calcium Sulfate

The solubility of the calcium sulfate sample was evaluated based on the amount of dissolved Ca^2+^ ions (g/L) with an increasing calcium/H_2_O ratio. The solubility was defined as the maximum amount of material dissolved in a liquid. The unit adopted for the solubility was g/L.

The solubility test was performed with increasing additions of calcium sulfate to 1 L of distilled water and the following concentrations were used: 2, 4, 6, 8, and 10 g/L. Test were performed at *T* = 22 ± 2 °C in an open system using a hydrometer. After shaking, the particles in the system were allowed to settle. In order to verify the solubility over time, the dissolved Ca^2+^ ions were quantified after periods of 0, 3, 11, and 18 days. These periods were coded as P0, P3, P11, and P18. The samples were stirred with the help of plunger (15 min). The amount of Ca^2+^ solubilized was determined at different intervals of time by energy dispersive X-ray (EDX) spectrometry analysis (Model 700 HS, Shimadzu, Tokyo, Japan). Dissolved aliquots were extracted (volume equal to 4 mL), filtered using a 25 µm membrane, and immediately analyzed by energy dispersive X-ray fluorescence spectrometry (Model 700 HS, Shimadzu). A Mylar film was used for the sample cell. 

#### 2.2.4. Workability and Setting Time

Workability was measured using a slump cone. In this study, the fluidity index was obtained based on EN 12706—Test methods for hydraulic setting floor smoothing and/or levelling compounds. Determination of flow characteristics. Determination of flow characteristics. The measurements were taken immediately after mixing. The spread width in two perpendicular directions on a plate was measured without drops after the spread of SLU stopped. The value acquired was recorded as a flow value.

A test was also performed to evaluate the maintenance of consistency over 180 min. In this test, the flow value is determined at pre-determined intervals of 30 min, with an acceptable average value being greater than or equal to 240 mm. 

#### 2.2.5. Measurement of Setting Time Using a Vicat Needle

To evaluate the flow time of the SLU samples, a V funnel test was performed. For this test, a flow time of not more than 10 s is desirable. A V-funnel was used to determine the flow time of the mortar. According to EFNARC [38], shorter flow times indicate greater flowability. For self-consolidation compounds SCC, a flow time of 10 s is considered appropriate.

#### 2.2.6. Compressive Strength

In these experiments, compressive strength was measured according to the standard method NBR NM 12142:2010. The SLUs were prepared for compressive strength tests in prismatic molds (40 mm × 40 mm × 160 mm). After demolding, they were stored in a temperature-controlled room (20 ± 3 °C) and the strength was determined after 1, 7, and 28 days of curing. X-ray diffraction analysis was carried out after 28 days in power using an X-Pert system, with CuKa = 1.54 Å. The SEM analysis was performed with a Philips microscope (model XL30). 

#### 2.2.7. Degradation in Water

For the study on the degradation in water, a procedure based on [39] was used. Prismatic molds (40 mm × 40 mm × 160 mm) were cast for this test. After demolding, samples were stored in a temperature-controlled room (20 ± 3 °C) for 28 days. 

Prismatic mortar sample were cast (dimensions 40 mm × 40 mm × 160 mm). After 24 h samples were demold and cured. Two procedures were performed: immersion in water and immersion-drying. In the immersion test, the samples underwent 28 days of immersion in drinking water and the weight of the specimen was measured daily throughout the test period. After 28 days in water, the mortar samples were stored in a temperature-controlled room and then underwent the compressive strength test. For the immersion-drying test, the specimen was kept for 16 h in water and 8 h in an oven at 50 °C over a period of 28 days.

The degradation rate of mixtures (k), obtained from the compressive strength of the material after 28 days of curing (C_28days_) and after the immersion and immersion-drying tests (C_tests_), is calculated according to the following Equation (2):

(2) $$k=\frac{C_{28\mathrm{days}}-C_{\mathrm{tests}}}{C_{28\mathrm{days}}}$$

When **k** is greater than 0 (zero), it is considered that the structure of the material is degraded; when **k** is less than or equal to 0 (zero) it indicates that the material is resistant to the action of water or immersion-drying.

The analysis of both calcium sulfate samples and the SLUs after 28 days was carried out.

## 3. Results and Discussion

### 3.1. Properties of Calcium Sulfate Samples: Phosphogypsum (PG REF), β-Hemihydrate, and Phosphoanhydrite (PA)

**Figure 1:**
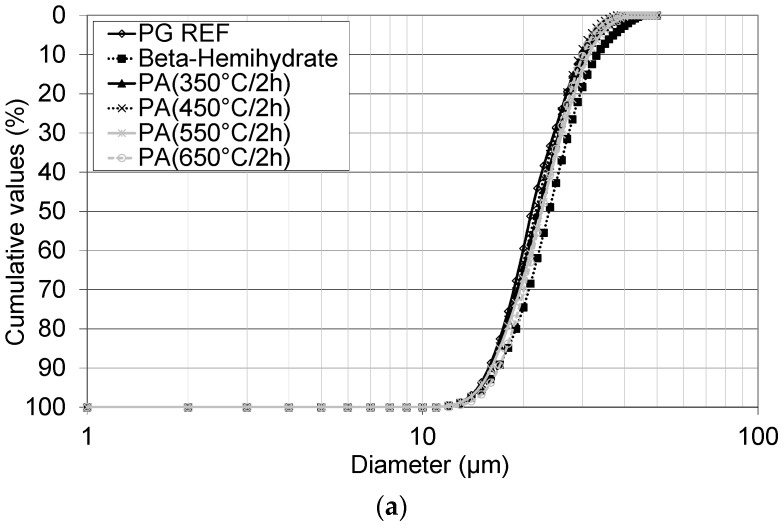

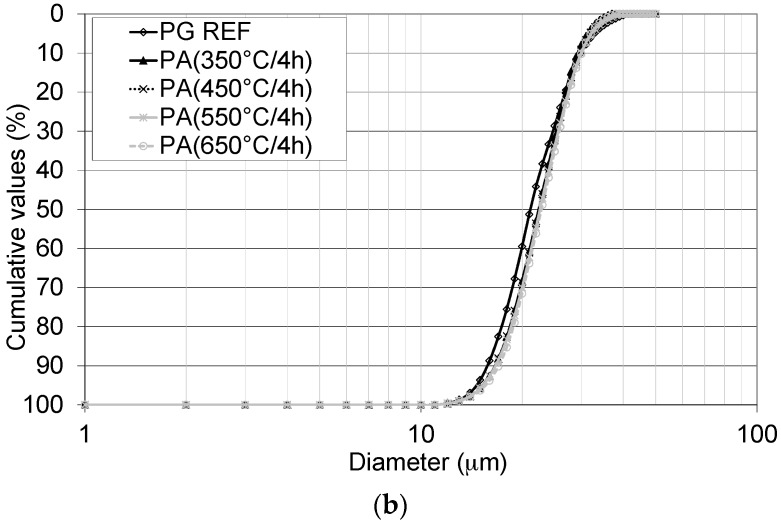
Particle size analysis of calcium sulfate samples studied: phosphogypsum (PG REF), β-hemihydrate, and phosphoanhydrite (PA) obtained from the calcination of gypsum at temperatures of 350 °C, 450 °C, 550 °C, and 650 °C during 2 h (**a**) and 4 h (**b**).

As seen in Table 5, the average diameter (D_50_) of phosphoanhydrite (PA) is approximately 34 µm. The samples analyzed in this study were composed of fine particles (<100 mm), as shown in Figure 1. As can be seen from the different curves there are no significant differences between the calcium sulfate samples.

**Figure 2:**
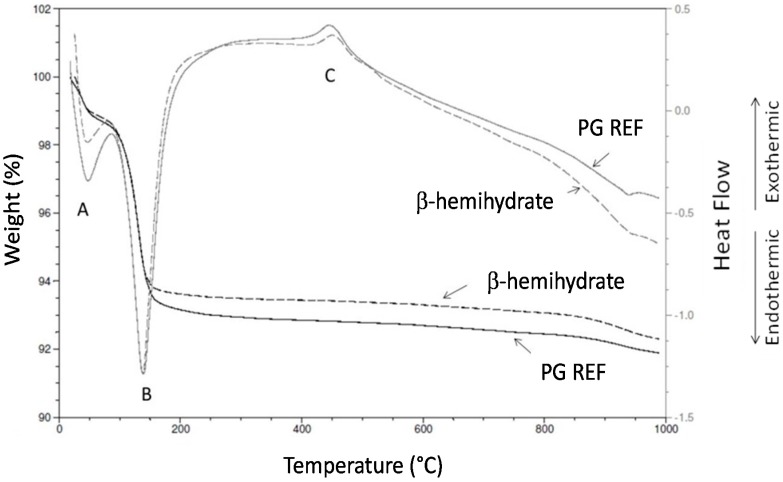
Thermal analysis of phosphogypsum (PG REF) and β-hemihydrate.

The comparison of thermograms (TG) curve shows a greater weight loss for phosphogypsum (PG REF) in relation to β-hemihydrate, which is due to a higher water content in the former, Figure 2. The weight loss was observed within two temperature ranges with 1.5% and 1.4% occurring at 23–50 °C and 5.2% and 4.8% at 100–150 °C, for PG REF and β-hemihydrate, respectively.

The dehydration stage at point A (50°) represents the evaporation of free water. Another endothermic peak observed at point B (150°) indicates the loss of water present in the crystal structure of phosphogypsum and β-hemihydrate. Point C (450°) corresponds to the crystalline transition of anhydrite III to anhydrite II. An endothermic event occurs with a low weight at close to 950 °C for the PG REF and β-hemihydrate samples. This thermal event could be associated with the phosphate rock (raw material) used in the P-fertilizer. According to [29] in phosphogypsum, P_2_O_5_ generally exists as H_3_PO_4_, Ca(H_2_PO_4_)_2_H_2_O, CaHPO_4_2H_2_O, and Ca_3_(PO_4_)_2_, P_2_O_5_ adhered at the surface of gypsum crystals and is water-leachable, and in the interstice of agglomerated crystal [23,40,41].

The materials have similar characteristics and it can be observed that to obtain phosphoanhydrite (anhydrite II) the temperature needs to be higher than 400 °C.

**Figure 3:**
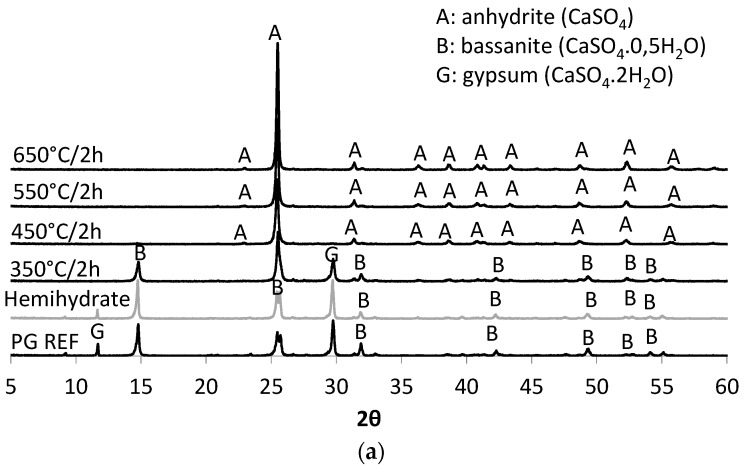

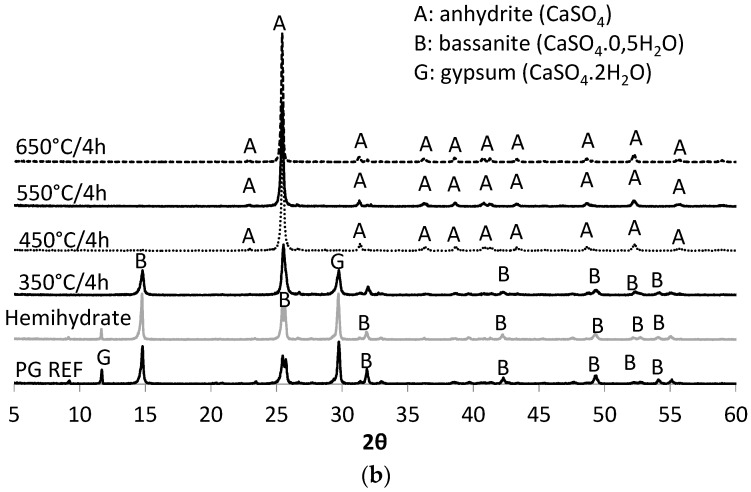
X-ray diffraction (XRD) diffractograms for phosphogypsum (PG REF), β-hemihydrate, and PAs. (**a**) Calcined for 2 h (**b**) calcined for 4 h.

The main crystalline compounds detected by X-ray diffraction in phosphogypsum (PG REF), β-hemihydrate, and phosphoanhydrite (PA) obtained from the calcination of the phosphogypsum at temperatures of 350 °C, 450 °C, 550 °C, and 650 °C for periods of 2 h and 4 h, are shown in Figure 3a and Figure 3b, respectvely. In N_2_ atmosphere, the main compounds present in the calcium sulfate samples were: gypsum (CaSO_4_·2H_2_O), bassanite (CaSO_4_·0.5H_2_O), and anhydrite (CaSO_4_).

As revealed by TG-DTA and demonstrated by XRD, the PA obtained at a temperature of 450 °C has clearly evident peaks associated with anhydrite at 2θ (theta) 25.43°, 40.23°, 43.03°, and 51.87°. The higher the calcination temperature, the more pronounced the anhydrite peaks (counts intensity) were, as can be seen in Table 6. At temperatures below 450 °C, peaks typical of gypsum and bassanite were recorded. It can be observed that the higher the temperature used in the calcination of the calcium sulfate, the greater the amount of ettringite formed, ensuring a greater strength at the age of one day. Based on ternary systems produced with anhydrite it was observed that a maximum quantity of ettringite is reached after one day, while for samples produced with β-hemihydrate the greatest occurrence of ettringite is observed after 3 h [17].

In relation to the period of calcination, it is clear, especially in the case of the PAs obtained at temperatures above 450 °C, that higher intensity peaks appeared when the samples were calcined for a longer period (4 h). Thus, the study on the SLUs was conducted with PAs obtained applying a calcination time of 4 h, with the exception of the degradation tests, which, as will be described below, were also carried out on PAs produced with 2 h of calcination.

**Figure 4:**
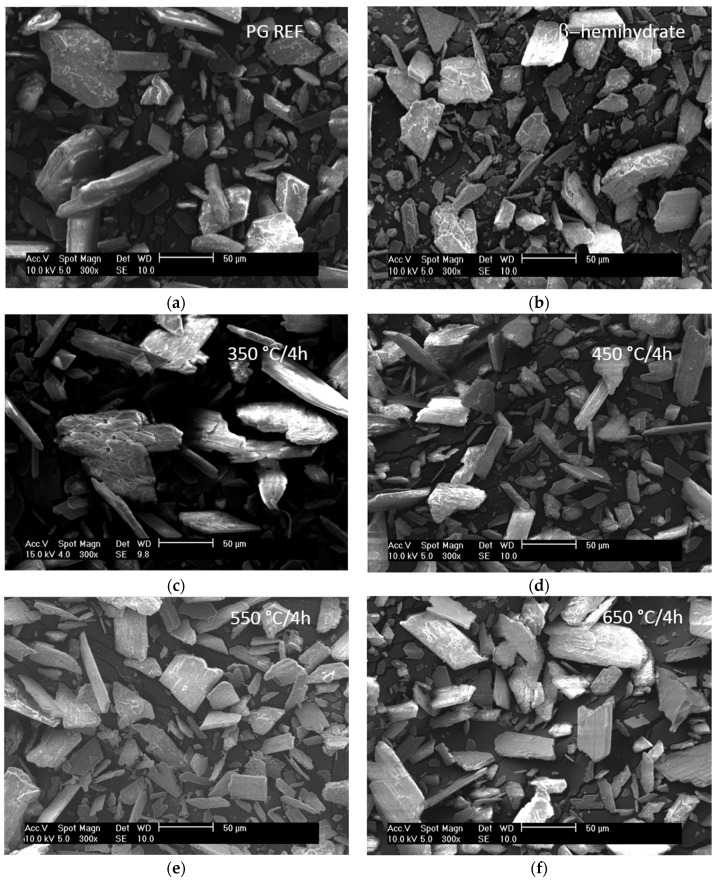
Morphology of calcium sulfate samples: phosphogypsum (PG REF) (**a**); β-hemihydrate (**b**); and PAs obtained at temperatures of 350 °C (**c**); 450 °C (**d**); 550 °C (**e**); and 650 °C (**f**).

Figure 4 shows the crystals formed in the PAs obtained at different heating temperatures (350 °C/4 h; 450 °C/4 h, 550 °C/4 h, 650 °C/4 h), the dried phosphogypsum (PG REF) and the β-hemihydrate.

The crystals of the β-hemihydrate and PG REF are pseudo-amorphous, Figure 4a and Figure 4b, respectively. Pseudo-amorphous crystals can be assigned to soluble anhydrite crystals, which are converted into anhydrite II with increasing calcination temperature.

When temperatures of 550 °C and 650 °C were applied, prismatic euhedral crystals with sharp edges were observed, Figure 4e and Figure 4f, respectively. These sharp edges allow better entanglement between the crystals that form when the phosphoanhydrite is added with other binders and water.

It also follows that the higher the calcination temperature, the larger the size and the greater the amount of prismatic crystals will be.

The formation of prismatic euhedral crystals with sharp edges is important to obtaining the best performance in terms of the compressive strength of mortars, providing more links between the hydrated products and acting as nucleation points. Impurities content in phosphogypsum could affect the nucleation points. 

### 3.2. Solubility of Calcium Sulfate

**Figure 5:**
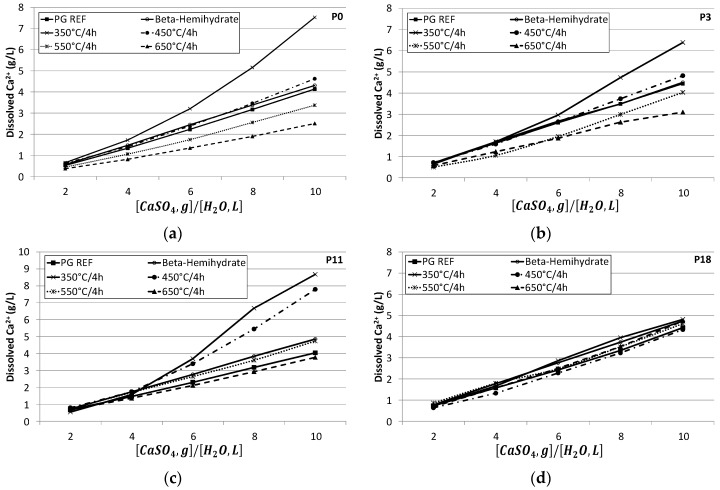
Solubility (g/L) over time. (**a**) Period = 0; (**b**) Period = 3 days; (**c**) Period = 11 days; (**d**) Period = 18 days.

Figure 5 shows that the higher the CaSO_4_/H_2_O ratio, the greater the amount of calcium which was dissolved. This is due to the greater availability of calcium sulfate in aqueous medium as the CaSO_4_/H_2_O ratio increases.

As the calcination temperature applied to obtain the PAs was increased, the dissolution in aqueous medium became slower. The dissolution of Ca^2+^ ions in the PA 550 °C/4 h and PA 650 °C/4 h samples occurs slowly and gradually as their concentration increases over time, reaching the level of dissolution observed for the calcium sulfate samples studied over 18 days (Figure 5d). Thus, it appears that these PA samples obtained at high temperature undergo slower hydration and are effectively anhydrite II, which is slightly soluble.

For the PA samples (350 °C/4 h) and (450 °C/4 h), note that the dissolution is initially high, in agreement with the XRD results, because despite the very clear anhydrite peaks (Figure 3), these samples are fairly soluble, as seen in the solubility test (Figure 5). 

This test was conducted in the dissolution direction. It is difficult to determine the exact solubility of hemihydrate since the gypsum can crystalize before the dissolution of the hemihydrate is complete. It is known that the solubility of α-hemihydrate, which is more crystallized and has a lower amount of defects, is inferior to that of β-hemihydrate. The solubility test in water was carried out in order to verify if the anhydrite produced in the calcination of the phosphogypsum has low solubility in water, this being a characteristic of type II anhydrite.

The higher the calcination temperature used to produce the Pas, the slower the dissolution in aqueous medium will be. The dissolution of the Ca^2+^ ions in the PAs 550 °C/4 h and 650 °C/4 h samples occurs slowly and gradually, since their concentration increases over time, equaling the dissolution of the other calcium sulfates studied over the period. For the PAs 350 °C/4 h and 450 °C/4 h, it was noted that the dissolution is already high in the initial periods. This complements the XRD results, since besides the evident peaks for anhydrite (Figure 3), this is very soluble, as verified in the solubility test. 

Considering only the dissolution of Ca2^+^ in P0 and in the highest CaSO_4_/H_2_O ratio, Table 7 shows the solubility of the calcium sulfate samples studied. In absence of anhydrite seeds, gypsum phases remained stable for periods of up to 18 days. At this period there is a complete transformation of the anhydrate and the solubility curves (Figure 5d) are similar. These results are in accordance with solubility diagrams modeled and presented by [41].

### 3.3. Properties of Calcium Sulfate-Based SLUs 

#### 3.3.1. Workability and Setting Time

A Vicat needle was used to determine the setting time during hardening (Table 8). 

A key feature of SLUs is their high fluidity. The SLUs produced provided consistency index values in the range of 270–310 mm, as shown in Table 8. These values are higher than the range suggested by [1] of 250–270 mm (flow value), but no segregation of the material was observed. 

The flow time measured by the funnel was above the established limit, however, the results were considered to be acceptable since the mortars remained cohesive and did not exceed the limit by more than two seconds.

The consistency index values for SLUs PA 550 °C/4 h and PA 650 °C/4 h were lower, and this behavior is due to the higher Blaine specific surface area of the PAs produced at these temperatures, requiring greater amounts of water (Table 1). To maintain the same water/binder ratio in all mortars it was necessary to increase the addition of superplasticizer, as shown in Table 4. These SLUs had longer setting times and, according to other authors [17], the hydration of anhydrite obtained at a high calcination temperature is slow.

The initial setting time for all formulations is short, around 49 min, which is similar to results obtained in a study on ettringite systems [24]. 

**Figure 6:**
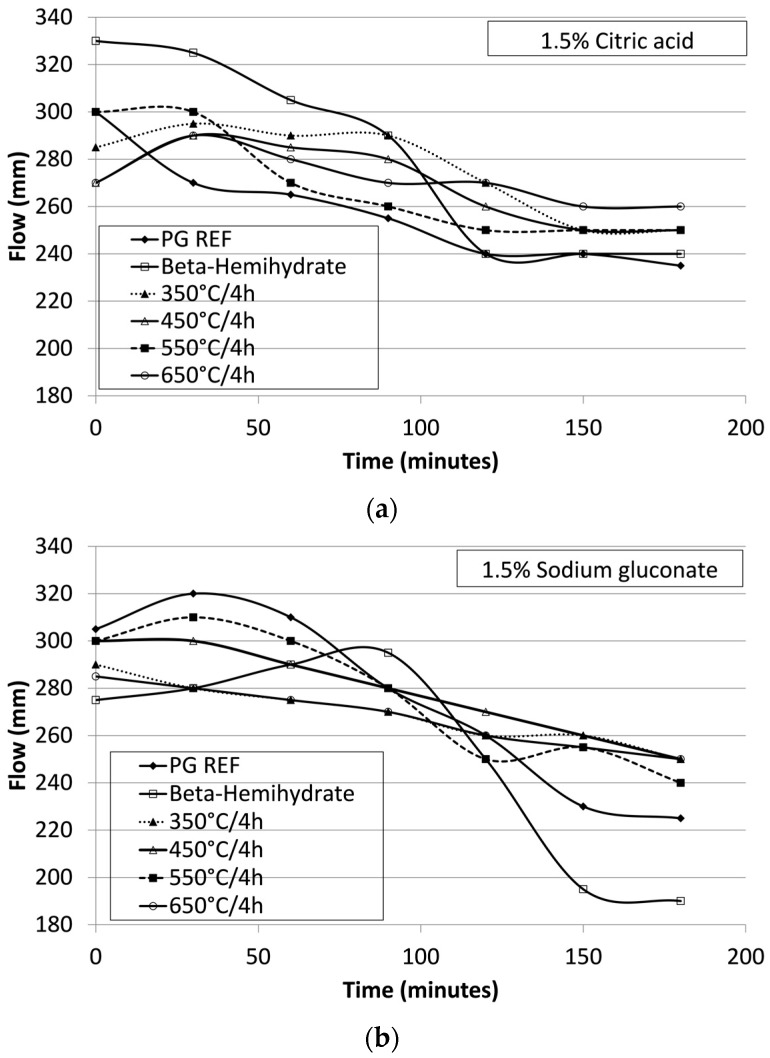
Maintenance of workability for 180 min with retardants citric acid (**a**) and sodium gluconate (**b**).

In order to maintain the workability for 180 min, the retardants citric acid and sodium gluconate were studied, Figure 6a and Figure 6b, respectively. Citric acid is most efficient in mixtures based on calcium sulfate [9], as verified in the test performed and shown in Figure 6a.

It was observed that when using citric acid, the workability, with a flow value of 250 mm, was maintained for 180 min, Figure 6a. An important observation is that the mechanical strength of the mortars was not altered by the addition of the retardant liquid. Also, it can be seen that, at the same concentration (1.5%), the sodium gluconate reduced a flowability after 90 min, Figure 6b.

#### 3.3.2. Compressive Strength and Degradation in Water of SLUs

**Figure 7:**
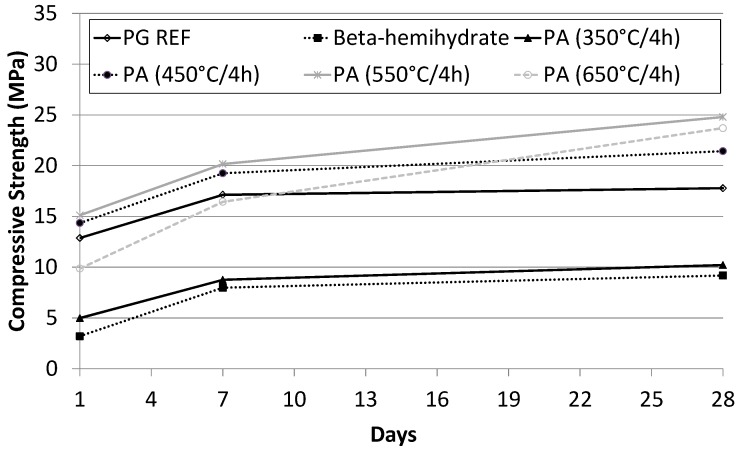
Compressive strength of SLU samples over 28 days.

The compressive strength values for self-leveling underlayments (SLU) at ages of 1, 7, and 28 days are given in Figure 7. The values refer to the arithmetic average of the results for 6 samples.

It appears that there was no significant increase in the compressive strength after 7 days of curing. After 28 days, higher mechanical strengths were obtained for the SLU samples PA 650 °C/4 h and PA 550 °C/4 h, with values greater than 23 MPa. The β-hemihydrate and PA 350 °C/4 h SLU samples showed the lowest compressive strength (10 MPa). 

The high strength after a shorter time can be attributed to the formation of ettringite in the first few hours. 

**Figure 8:**
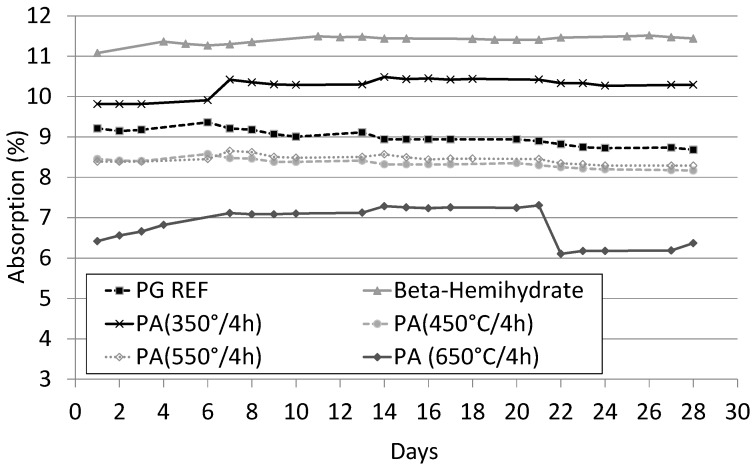
Absorption of water by SLUs during the immersion test.

Figure 8 shows the behavior of the SLUs in water, observed through their daily mass values obtained during the immersion test. The self-leveling mortar with the highest water absorption (11.1%) was that produced with β-hemihydrate. This was followed by PA 350 °C/4 h which absorbed around 10%. This may indicate a higher porosity in relation to the other samples, as these mortars had lower mechanical strength.

The self-leveling underlayment PA 650 °C/4 h showed the lowest absorption. However, a variation in its mass over time was observed during the immersion test, this being affected by the weekly renewal of water in the test vessel, a little precipitation was observed. This indicates the possible solubility of the Ca^2+^ and SO^4+^ ions and their loss from the porous medium during water immersion, resulting in a reduction in the sample mass. This change in the sample mass did not result in a decrease in compressive strength. The other SLUs studied showed stable behavior during the test.

The degradation coefficient (*k*) after immersion and immersion-drying cycles is shown in Table 9. It appears that the period of calcination used to obtain anhydrite II is of great importance. When the PA used in the production of SLU was calcined for 2 h, all formulations degraded, with k being greater than zero. This confirms the results obtained for the PAs in the XRD analysis (Figure 3).

The self-leveling mortars with PAs calcined for 4 h showed good resistance to degradation in water (Table 9). The microstructure of the material was damaged in the case of the SLU PA (450 °C/4 h), which reduced the compressive strength by 14% and 32% after the immersion test and immersion-drying, respectively.

Although the compressive strength of the SLU PA 450 °C/4 h was found to be satisfactory, this was the only sample which showed degradation. It should be noted that in the water degradation tests the compressive strength was measured while the mortars were still wet, leading to lower values. Thus, on comparing the calcination temperature and degradation in water for the mortars, it can be concluded that the calcination is satisfactory at temperatures of up to 650 °C, providing slightly soluble anhydrite (anhydrite II).

**Figure 9:**
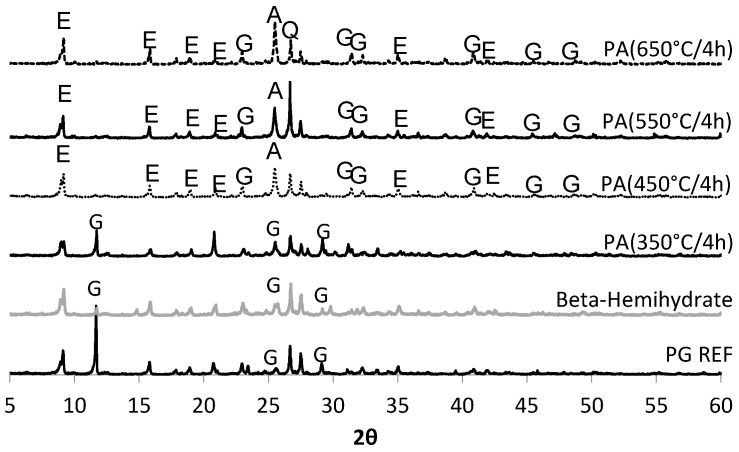
X-ray diffraction patterns for SLUs after 28 days of curing. E = Ettringite, G = Gypsum, A = Anhydrite, and Q = Quartz.

The formation of ettringite may contribute to the strength of the SLU samples, and to preventing the water-degradation after the immersion and immersion-drying cycles. The main crystalline product in the SLU samples PA 550 °C/4 h and PA 650 °C/4 h is ettringite, as can be observed in the XRD patterns in Figure 9. It can be observed that the peak intensity increases with the calcination temperature. The presence of ettringite was also observed in the mortars PG REF, β-hemihydrate, and PA 350 °C/4 h, however, the main crystalline component of these mortars is gypsum (calcium sulfate dihydrate). 

According to these results, it is suggested that the order of ettringite formation in systems rich in PG-CAC-PC is the following: PA 650 °C/4 h > PA 550 °C/4 h > PA 450 °C/4 h > PA 450 °C/4 h > β-hemihydrate > PG-REF.

**Figure 10:**
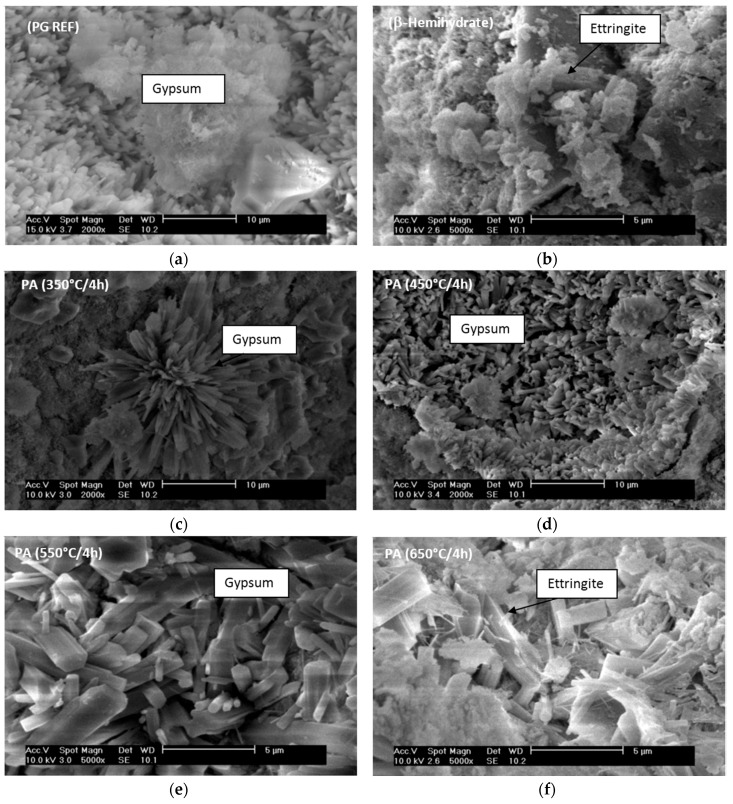
SEM images of self-leveling underlayments after 28 days of curing. (**a**) PG REF; (**b**) β-hemydrate; (**c**) PA 350 °C/4 h; (**d**) PA 450 °C/4 h; (**e**) PA 550 °C/4 h; (**f**) PA 650 °C/4 h.

SEM images show anhydrous gypsum present in the matrix in two morphologies: large plaques (Figure 10a) and tabulars (Figure 10c). Different morphologies of ettringite were observed, such as short needle-shaped (Figure 10b) and acicular or fine needle-shaped (Figure 10e). 

Some authors attribute the formation of fine needles of ettringite to the type of calcium sulfate used. In this study, the PA was obtained by calcination at a high temperature. The PA used undergoes slow hydration and the calcium aluminate present in cement solubilizes rapidly since the solution is poor in sulfate, leading to the growth of fine needles of ettringite. In addition, typical tabular crystals of gypsum were observed (Figure 10d–f).

## 4. Conclusions

The main conclusions of this study are:

- Applying a longer calcination time to the phosphogypsum used to obtain phosphoanhydrite (PA) increases its specific surface area.
- Regarding the phases formed, higher anhydrite peaks were obtained for higher calcination temperatures and also for a longer period of thermal retention. The XRD spectra and thermograms showed that to obtain anhydrite II for use as a binder, a temperature higher than 450 °C is required. SEM micrographs revealed euhedral crystals with sharp edges at temperatures of 550 °C and 650 °C, which contributes to the formation of nucleation sites in hydrated products.
- The solubility of calcium sulfate varies according to the calcination temperature and the higher the temperature, the less soluble the calcium sulfate will be.
- SLUs can be produced using phosphoanhydrite (PA) as the main binder, but the handling time is short, requiring the use of retarders. It was found that citric acid is more effective than sodium gluconate in terms of maintaining the consistency of systems with a predominance of calcium sulfate.
- Only the self-leveling mortar PA 450 °C/4 h degraded in water during immersion and immersion-drying tests. The formation of ettringite, detected by XRD, ensured that the PA sample was not sensitive to water.
- Different ettringite morphologies were observed by SEM for the SLUs after 28 days of curing. The morphology varied according to the type of calcium sulfate used in the production of the mortar sample, being short needle-shaped when sulfates of rapid solubilization, such as PG REF and β-hemihydrate, are used and fine needle-shaped when using phosphoanhydrite (PA), which hydrates slowly.

## Figures and Tables

**Table 1:** Physical properties of dried phosphogypsum (PG REF), β-hemihydrate, and phosphoanhydrite (PA).

Time Heating	Heating Temperature (°C)	Specific Gravity (Kg/m^3^)	Specific Surface Area (cm^2^/g)	Loss on Ignition (%)
Reference	PG REF	2570	1073	26
	β-hemihydrate	2640	1756	14
2 h	350 °C	2670	1259	5
		450 °C	2720	1379	1
		550 °C	2740	1458	3
		650 °C	2830	1465	11
4 h	350 °C	2650	1623	3
		450 °C	2770	1686	2
		550 °C	2790	1741	2
		650 °C	2840	1742	11

**Table 2:** Chemical analysis of dried phosphogypsum (PG REF), β-hemihydrate and phosphoanhydrite (PA).

Oxide wt (%)	PG REF	β-Hemihydrate	350 °C/4 h	450 °C/4 h	550 °C/4 h	650 °C/4 h
CaO	38.560	45.763	52.058	53.53	54.385	47.772
SO_3_	24.593	30.493	33.236	34.101	33.932	31.114
Na_2_O	3.993	2.336	4.84	3.85	3.694	3.396
BaO	1.362	1.747	0.468	0.511	0.576	1.236
Fe_2_O_3_	0.898	1.254	1.047	1.029	1.101	1.026
P_2_O_5_	1.130	1.326	1.396	1.011	1.460	1.238
SrO	0.856	0.937	1.126	1.136	1.182	1.012
SiO_2_	0.820	1.028	1.151	0.92	1.026	0.986
Al_2_O_3_	0.411	0.234	0.226	0.101	0.126	0.160
Cl	0.289	0.226	0.358	0.297	0.293	0.176
Sc_2_O_3_	0.948	0.535	0.915	1.300	ND	0.682
MnO	0.078	0.057	0.123	0.123	0.130	0.104
NbO	0.032	0.035	0.038	0.038	0.046	0.035
CO_2_	26	14	3	2	2	11
Others	0.03	0.029	0.018	0.053	0.049	0.063

ND = not determined.

**Table 3:** Chemical analysis of cements (Oxide wt %).

Cements	Al_2_O_3_	TiO_2_	SiO_2_	Fe_2_O_3_	ZrO_2_	MgO	CaO	Na_2_O	K_2_O	* SSA (cm^2^/g)
CAC	60.32	0.51	4.09	0.36	-	0.14	34.39	0.15	0.04	3877
PC	4.09	-	18.51	2.58	-	4.95	59.83	-	-	3223

* Specific surface area (Blaine).

**Table 4:** Percentage of raw materials in SLU (%).

Materials (wt %)	PG REF	β-Hemihydrate	350 °C	450 °C	550 °C	650 °C
Quartz sand	67	67	67	67	67	67
CAC	8.25	8.25	8.25	8.25	8.25	8.25
PC	4.95	4.95	4.95	4.95	4.95	4.95
FG	19.80	-	-	-	-	-
β-Hemihydrate	-	19.80	-	-	-	-
PA	-	-	19.80	19.80	19.80	19.80
Superplasticizer (%)	1.15	1.15	1.3	1.3	1.3	1.3

**Table 5:** Particle size (D_10_ and D_50_).

Heating Time	Calcium Sulfate	D_10_ (µm)	D_50_ (µm)
Reference	PG REF	9.3	42.8
	β-Hemihydrate	5.3	20.3
2 h	350 °C	8.3	36.1
	450 °C	10.1	38.5
	550 °C	7.3	33.1
	650 °C	9.1	32.7
4 h	350 °C	10.5	34.5
	450 °C	9.7	34.0
	550 °C	9.3	33.3
	650 °C	9.1	32.0

**Table 6:** Major peaks in the formation of anhydrite from the calcination of phosphogypsum.

		Peak Position	(2θ)		
Heating Time	T (°C)	25.43°	40.23°	43.03°	51.87°
4 h	350 °C	535	13	20	11
	450 °C	1540	9	17	14
	550 °C	1886	6	10	14
	650 °C	2316	10	13	6
2 h	350 °C	647	7	11	6
	450 °C	1280	7	15	8
	550 °C	1706	10	17	7
	650 °C	1900	4	12	11

**Table 7:** Solubility of each calcium sulfate sample.

Calcium Sulfate	Solubility (g/L)
PG REF	4.1
β-Hemihydrate	4.3
350 °C/4 h	7.5
450 °C/4 h	4.6
550 °C/4 h	3.3
650 °C/4 h	2.5

**Table 8:** Workability and setting time of SLUs.

Mortar SLU Design	Flow Value (Diameters)	Flow Time (V Funnel)	Setting Time (h:min)		
	mm	Seconds	Initial	Final	∆time
PG REF	303	10	00:43	01:15	00:32
β-Hemihydrate	304	10	00:32	00:59	00:27
350 °C/4 h	310	11	00:49	01:13	00:24
450 °C/4 h	306	12	00:30	00:56	00:26
550 °C/4 h	270	12	00:42	01:35	00:53
650 °C/4 h	270	12	00:41	01:31	00:50

**Table 9:** Compressive strength of self-leveling underlayments after 28 days (C_28days_) and after 28 days of immersion (C_immersion_) and immersion-drying (C_im-drying_) cycles.

Mortars SLU Design	C_28days_	C_immersion_	K	C_im-drying_	K
PG REF	17.80	19.31	−0.08	18.51	−0.04
β-Hemihydrate	9.20	9.20	0.00	9.21	0.00
350 °C/4 h	10.20	11.31	−0.11	10.36	−0.02
450 °C/4 h	21.40	18.40	0.13	14.60	0.32
550 °C/4 h	24.80	26.96	−0.08	25.64	−0.03
650 °C/4 h	23.70	23.86	−0.01	25.28	−0.07
350 °C/2 h	17.90	11.00	0.39	10.36	0.42
450 °C/2 h	21.80	15.50	0.29	14.60	0.33
550 °C/2 h	20.70	17.87	0.14	19.64	0.05
650 °C/2 h	21.09	19.57	0.07	20.28	0.04

## References

[1] Georgin J.F., Ambroise J., Péra J., Reynouard J.M. (2008). Development of self-leveling screed based on calcium sulfoaluminate cement: Modelling of curling due to drying. Cem. Concr. Compos..

[2] Canbaz M., Topçu I.K., Ateşin O. (2016). Effect of admixture ratio and aggregate type on self-leveling screed properties. Constr. Build. Mater..

[3] Emoto T., Bier T.A. (2007). Rheological behavior as influenced by plasticizers and hydration kinetics. Cem. Concr. Res..

[4] Le-Bihan T., Georgin J.F., Michel M., Ambroise J., Morestin F. (2012). Measurements and modeling of cement base materials deformation at early age: The case of sulfo-aluminous cement. Cem. Concr. Res..

[5] Odler I. (2000). Water resistant gypsum binder. Special Inorganic Cements. Modern Technology.

[6] Bizzozero J., Scrivener K.L. (2015). Limestone reaction in calcium aluminate cement–calcium sulfate systems. Cem. Concr. Res..

[7] Xu L., Wang P., Zhang G. (2012). Formation of ettringite in Portland cement/calcium aluminate cement/calcium sulfate ternary system hydrates at lower temperatures. Constr. Build. Mater..

[8] Singh N.B., Middendorf B. (2007). Calcium sulphate hemihydrate hydration leading to gypsum crystallization. J. Prog. Cryst. Growth Charact. Mater..

[9] Onishi K., Bier T.A. (2010). Investigation into relations among technological properties, hydration kinetics and early age hydration of self-leveling underlayments. Cem. Concr. Res..

[10] Tosun K., Baradan B. (2010). Effect of ettringite morphology on DEF related expansion. Cem. Concr. Compos..

[11] Evju C., Hansen S. (2005). The kinetics of ettringite formation and dilatation in a blended cement with hemihydrate and anhydrite as calcium sulfate. Cem. Concr. Res..

[12] Kighelman J., Scrivener K. (2008). Kinetics of two types of flooring mortar: PC dominated vs CAC dominated. Calcium Aluminate Cements, Proceedings of the Centenary Conference, Avignon, France, 30 June–2 July 2008.

[13] Qoku E., Bier T.A., Westphal T. (2017). Phase assemblage in ettringite-forming cement pastes: A X-ray diffraction and thermal analysis characterization. J. Build. Eng..

[14] Ambroise J., Pera J. (2008). Development of self-levelling screed based on calcium aluminate. Calcium Aluminate Cements, Proceedings of the Centenary Conference, Avignon, France, 30 June–2 July 2008.

[15] Kighelman J. (2007). Hydration and Structure Development of Ternary Binder System as Used in Self-Levelling Compounds. Ph.D. Thesis.

[16] Smair M. (2008). Ternary system: Calcium aluminate Portland-cement-gypsum. Calcium Aluminate Cements, Proceedings of the Centenary Conference, Avignon, France, 30 June–2 July 2008.

[17] Sievert T., Wolter A., Singh N.B. (2005). Hydration of anhydrite of gypsum (CaSO4.II) in a ball mill. Cem. Concr. Res..

[18] Hincapie A.M., Cincotto M.A. (1997). Efeitos de retardadores de pega no mecanismo de hidratação e na microestrutura do gesso de construção. Ambiente Construído.

[19] Singh M., Garg M. (1995). Activation of gypsum anhydrite-slag mixtures. Cem. Concr. Res..

[20] Murat M. Structure cristallochimie et réactivité des Sulfates de Calcium. Proceedings of the UIn: U Compterendu du Colloque International de la RILEM.

[21] Camarini G., Milito J.A. (2011). Gypsum hemihydrate-cement blends to improve renderings durability. Constr. Build. Mater..

[22] Canut M.M.C., Jacomino V.M.F., Bratveit K., Gomes A.M., Yoshida M.I. (2008). Microstructural analyses of phosphogypsum generated by Brazilian fertilizer industries. Mater. Charact..

[23] Singh M., Garg M. (2000). Making of anhydrite cement from waste gypsum. Cem. Concr. Res..

[24] Duan Z., Li J., Li T., Zheng S., Han W., Geng Q., Gu H. (2017). Influence of crystal modifier on the preparation of α-hemihydrate gypsum from phosphogypsum. Constr. Build. Mater..

[25] Lanzón M., García-Ruiz P.A. (2011). Effect of citric acid on setting inhibition and mechanical properties of gypsum building plasters. Constr. Build. Mater..

[26] Lamberet S., Amathieu L., Scrivener K.L. (2008). Microstructure development of binder based on calcium aluminate cement, calcium sulfate and Portland cement. Calcium Aluminate Cements, Proceedings of the Centenary Conference, Avignon, France, 30 June–2 July 2008.

[27] Zhang H., Lin Z., Tong D. (1996). Influence of the type of calcium sulfate on the strength and hydration of Portland and Cement under an initial steam-curing condition. Cem. Concr. Res..

[28] (2017). Environmental assessment and management of Phosphogypsum according to European and United States of America regulations. Procedia Earth Planet. Sci..

[29] Tayibi Y., Choura H., López M., Alguacil F.A., Delgado A.L. (2009). Enviromental impact and management of phosphogypsum. J. Environ. Manag..

[30] Silva N.C., Fernandes E.A.N., Cipriani M., Taddei1 M.H.T. (2001). The natural radioactivity of Brazilian phosphogypsum. J. Radioanal. Nucl. Chem..

[31] Borges R.C., Ribeiro F.C.A., da Costa Lauria D., Bernedo A.V.B. (2013). Radioactive characterization of phosphogypsum from Imbituba Brazil. J. Environ. Radioact..

[32] Rutherford P.M., Dudas M.J., Arocena J.M. (1995). Radioactivity and elemental composition of phosphogypsum produced from three phosphate rock sources. Waste Manag. Res..

[33] Maringer F.J., Baumgartner A., Rechberger F., Seidel C., Stietka M. (2013). Activity measurement and effective dose modelling of natural radionuclides in building material. Appl. Radiat. Isot..

[34] Maduar M.F., Campos M.P., Mazzilli B.P., Villaverde F.L. (2011). Assessment of external gamma exposure and radon levels in a dwelling constructed with phosphogypsum plates. J. Hazard. Mater..

[35] Kovler K. (2017). The national survey of natural radioactivity in concrete produced in Israel. J. Environ. Radioact..

[36] EC 13—COUNCIL DIRECTIVE 2013/59/EURATOM. https://ec.europa.eu.

[37] Campos M.P., Costa L.J.P., Nisti M.B., Mazzilli B.P. (2017). Phosphogypsum recycling in the building materials industry: Assessment of the radon exhalation rate. J. Environ. Radioact..

[38] EFNARC SCC (2002). Specification and Guidelines for Self-Compacting Concrete.

[39] Kuryatnyk T. (2007). Insensibilisation à l’eau des Misturas à Base de Sulfate de Calcium par Ajout de Clinker Sulfo-Alumineux. Ph.D. Thesis.

[40] Singh M. (2002). Treating waste phosphogypsum for cement and plaster manufacture. Cem. Concr. Res..

[41] Azimi G. (2010). Evaluation the Potential of Scaling Due to Calcium Compounds in Hydrometallurgics Process. Ph.D. Thesis.

